# Endodontic Procedural Errors: Frequency, Type of Error, and the Most Frequently Treated Tooth

**DOI:** 10.1155/2015/673914

**Published:** 2015-08-10

**Authors:** Waqas Yousuf, Moiz Khan, Hasan Mehdi

**Affiliations:** ^1^Department of Oral Surgery, Fatima Jinnah Dental College and Hospital, Karachi, Sindh, Pakistan; ^2^Departments of Oral Pathology and Oral Surgery, Fatima Jinnah Dental College and Hospital, Karachi, Sindh, Pakistan

## Abstract

*Introduction.* The aim of this study is to determine the most common endodontically treated tooth and the most common error produced during treatment and to note the association of particular errors with particular teeth. *Material and Methods.* Periapical radiographs were taken of all the included teeth and were stored and assessed using DIGORA Optime. Teeth in each group were evaluated for presence or absence of procedural errors (i.e., overfill, underfill, ledge formation, perforations, apical transportation, and/or instrument separation) and the most frequent tooth to undergo endodontic treatment was also noted. *Results.* A total of 1748 root canal treated teeth were assessed, out of which 574 (32.8%) contained a procedural error. Out of these 397 (22.7%) were overfilled, 155 (8.9%) were underfilled, 16 (0.9%) had instrument separation, and 7 (0.4%) had apical transportation. The most frequently treated tooth was right permanent mandibular first molar (11.3%). The least commonly treated teeth were the permanent mandibular third molars (0.1%). *Conclusion.* Practitioners should show greater care to maintain accuracy of the working length throughout the procedure, as errors in length accounted for the vast majority of errors and special care should be taken when working on molars.

## 1. Introduction

Bacterial elimination from the root canal system holds the key to a successful endodontic treatment [1]. The primary determinant to achieve this and to prevent future encroachment of bacteria is a thorough and meticulous technique. When these measures are taken into account success rate has been shown to be as high as 94% [2, 3]. The proper technique becomes of particular importance in presence of periapical infection. This was well demonstrated in a study by Chugal et al., who showed that, for every 1 mm loss of working length, in teeth with apical periodontitis, failure rate increases by 14% [4].

Poor technique can be manifested in numerous ways. These include errors in length (i.e., overfill and underfill), errors in cleaning and shaping (i.e., ledge formation, apical transportation, perforations, and instrument fracture), and errors in quality of obturation (i.e., voids, lack of uniform and continuous taper, and lack of homogeneity). Presence of such errors can produce dire consequences.

Certain errors have undoubtedly been revealed to have a significantly negative impact on the final outcome. Underfill has been shown to reduce success rate to a mere 68% [5–7]. Similarly, overfill also contributes to failure and has been shown to reduce success rate to as low as 76% [6–8]. Instrument separation has also been shown to reduce the success rate by up to 14% when compared to those in which there was no instrument separation [5, 9]. However, in this case percentage of failure depends on the degree of debridement that was achieved prior to instrument separation.

The aim of this study is to determine the most common endodontically treated tooth and the most common error produced during treatment and to note the association of particular errors with particular teeth. This will help practitioners to determine which steps of the endodontic procedure requires greater diligence, in order to substantially improve the quality of their work and ensure better long term viability of the treatment.

## 2. Material and Methods

### 2.1. Study Design

This is a retrospective study.

### 2.2. Setting

The study was carried out in Fatima Jinnah Dental Hospital.

### 2.3. Sample Size

Sample size is 1748.

### 2.4. Purposive Sampling

Teeth treated with both conventional and rotary filing systems by postgraduate trainees from 2011 to 2014 were recruited into this study.

### 2.5. Inclusion Criteria

Inclusion criteria are as follows: (1) patients aged between 12 years and 65 years, (2) all permanent maxillary and mandibular teeth, (3) teeth prepared with conventional stainless steel files, and (4) root canal treatment performed by postgraduate trainees.

### 2.6. Exclusion Criteria

Exclusion criteria are as follows: (1) teeth with open apices, (2) teeth with blocked canals, (3) external root resorption, (4) lateral root resorption, (5) periapical pathology (such as cysts and tumors), and (6) advanced periodontal conditions/perio-endo lesions.

### 2.7. Data Collection

All the root canal treated teeth that fulfilled the inclusion criteria were included in this study, after approval by the Institutional Ethical Review Committee. All teeth treated with conventional files were prepared using the crown down technique and were obturated using lateral condensation technique. Crown down technique was performed using conventional stainless steel hand files. Coronal flaring was initially done using Gates-Glidden burs. K-files were used to shape the canals in the following sequence: #55, #50, #45, #40, #35, and #30. Size 30 was taken as the master apical file (MAF). Working length was deemed acceptable if it was within 0–2 mm of the radiographic apex as determined by a periapical radiograph taken using a paralleling technique. For the purposes of our study overfill was defined as extrusion of root canal filling material (gutta-percha) beyond the radiographic apex. Underfill was defined as root canal filling material (gutta-percha) more than 2 mm short of the radiographic apex. Instrument separation was defined as when instrument fracture occurred at any point during the procedure and was irretrievable. Apical transportation was defined as undesirable deviation from the normal canal path. Dr. Herbert Schilder [10] in 1967 defined overextension and underextension of the root canal filling as solely the matter of its vertical dimension being beyond or short of the root apex. According to his definition the overfilled canal is one which was well filled in three dimensions but exhibited surplus filling material past the apex. The underfill root canal was defined as one which fails to fill the circumference of the apical foramen in one or more dimensions, leaving voids for stagnation of fluids, recontamination, and persistence of infection. However, as it was unfeasible to assess root canal treatments in three dimensions in our setting, the terms overfill and overextension as well as underfill and underextension are used interchangeably in this study.

The periapical radiographs (taken with a paralleling technique) of these teeth were stored in DIGORA Optime and were grouped into two categories:

1. treatment with a procedural error,
2. treatment without a procedural error.

Teeth in each group were evaluated for presence or absence of procedural errors (i.e., overfill, underfill, ledge formation, perforations, apical transportation, and/or instrument separation) and the most frequent tooth to undergo endodontic treatment was also noted.

Incidence of each individual type of error was calculated. Radiographs were assessed by two assistant professors and in case of difference in opinion the relevant X-ray was shown to the professor of the department and his opinion was taken as final.

### 2.8. Data Analysis

Data was analyzed using SPSS version 21. Chi-square test was used to test the *p* value.

## 3. Results

A total of 1748 root canal treated teeth were assessed, out of which 1059 (61.1%) belonged to females and 674 (38.9%) to males. However, this female predisposition was not statistically significant (*p* > 0.05).

**Figure 1:**
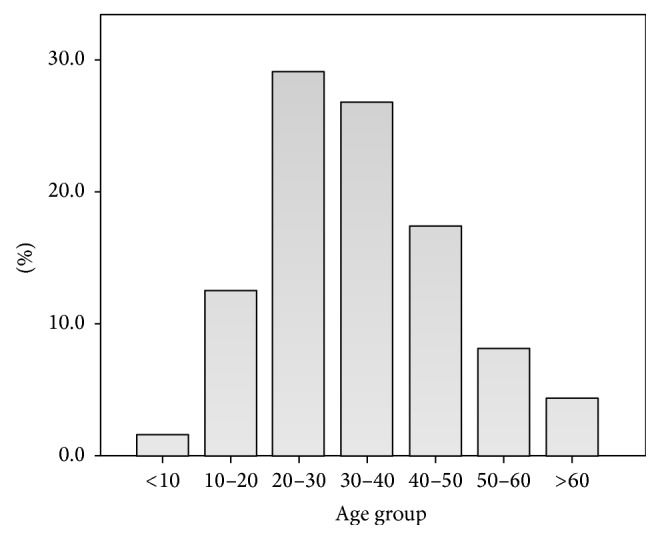
Age distribution of all root canal treatment cases.

Out of the total sample, 940 were maxillary teeth (53.8%) and 808 were mandibular teeth (46.2%). The mean age of the participants was 33.2 years ± 13.2. In males the mean age was 32.8 ± 14 and in females the mean age was 33.4 ± 12.3 (see Figure 1).

**Figure 2:**
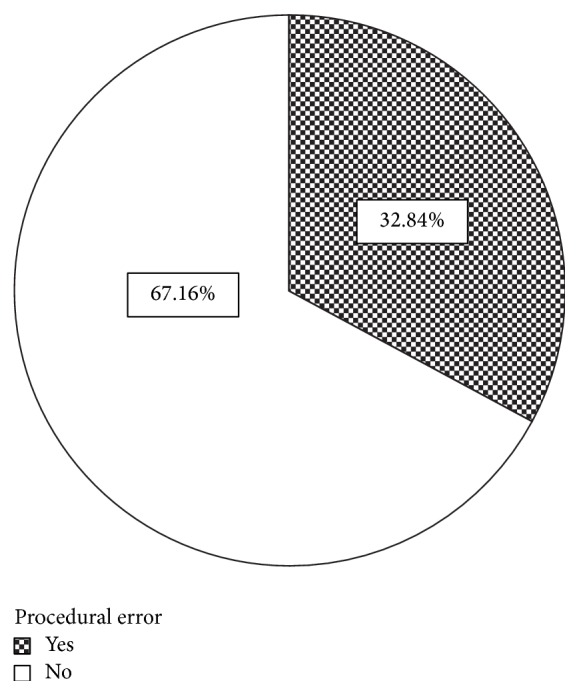
Overall procedural errors.

**Figure 3:**
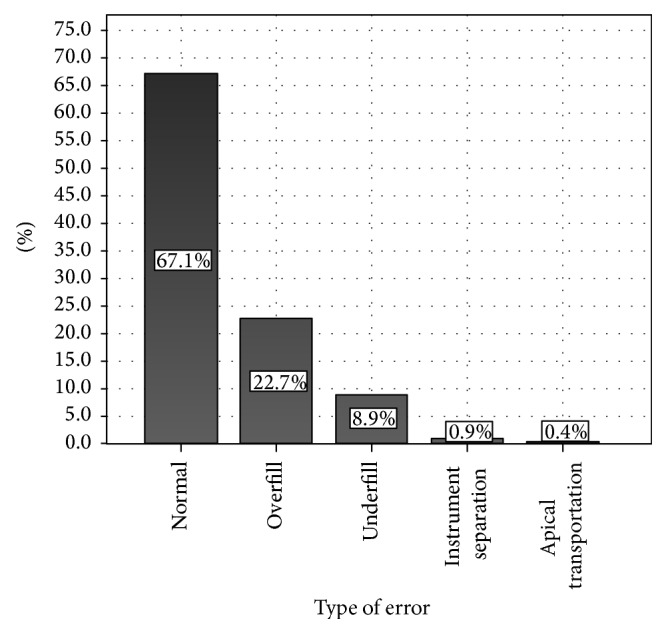
Distribution of procedural errors in all root canal treatment cases.

Out of the total number of cases, 574 (32.8%) contained a procedural error (see Figure 2), out of which 397 (22.7%) were overfilled, 155 (8.9%) were underfilled, 16 (0.9%) had instrument separation, and 7 (0.4%) had apical transportation (see Figure 3).

The most frequently treated tooth was the right permanent mandibular first molar (11.3%), followed by the left permanent mandibular first molar (10.0%), right permanent maxillary first molar (7.0%), and left permanent maxillary first molar (6.5%).

**Figure 4:**
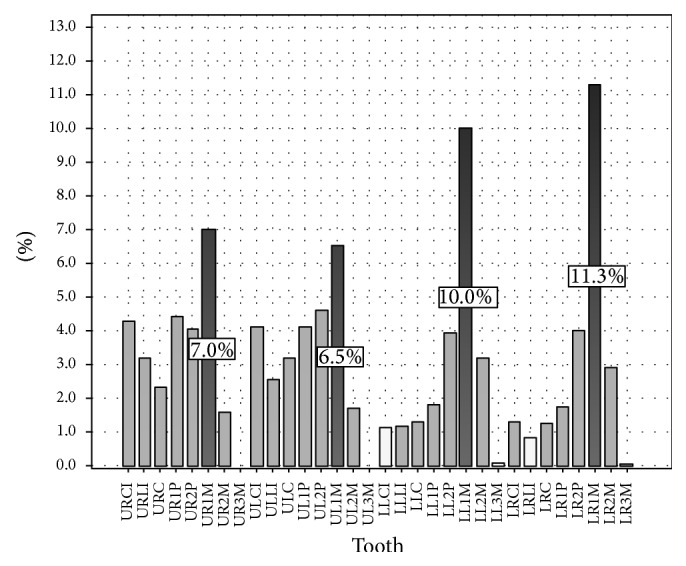
Distribution of endodontically treated teeth [UR: upper right, UL: upper left, LR: lower right, LL: lower left, CI: central incisor, LI: lateral incisor, C: canine, 1P: first premolar, 2P: second premolar, 1M: first molar, 2M: second molar, and 3M: third molar].

The least commonly treated teeth were the permanent mandibular third molars (0.1%), followed by the right permanent mandibular lateral incisor (0.9%) and left permanent mandibular central incisor (1.1%) (see Figure 4).

**Figure 5:**
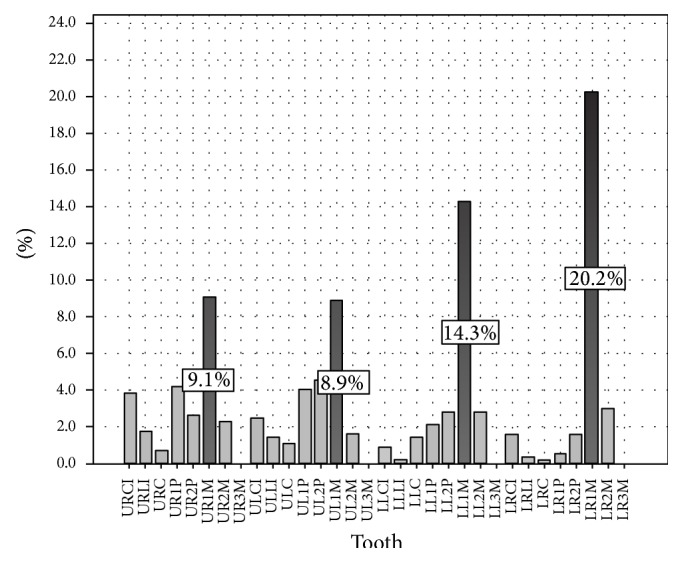
Distribution of teeth possessing a procedural error.

The most frequent tooth to possess an error was the right permanent mandibular first molar (20.2%), followed by the left permanent mandibular first molar (14.3%), right permanent maxillary first molar (9.1%), and left permanent maxillary first molar (8.9%) (see Figure 5).

No statistically significant association between gender and type of procedural error was observed. Overfill tends to occur more frequently in 10–20 years' age group, whereas underfill was less frequently observed in this age group compared to the other groups. However, after removing outlying groups (<10 and >60), these relationships were found to be statistically insignificant.

Similarly, underfill tends to occur more frequently in 50–60 years' age group, whereas overfill was less frequently observed in this age group compared to the other groups. However, after removing outlying groups (<10 and >60), these relationships were also found to be statistically insignificant (see Table 1).

Canines were the least affected by procedural errors (86.6% normal cases), followed by the incisors, which when compared to the canines had a much higher rate of overfill (17.4% versus 7.7%). However, this proved to be statistically insignificant. Molars were by far the most affected tooth group showing the greatest percentage of errors in each category and showing a meager 54.1% normal cases.

Underfill occurred more frequently in posterior tooth groups (premolars and molars) when compared to anterior tooth groups (incisors and canines). Instrument separation was seen more than twice as frequently in molars as compared to the next most frequent groups (canines) (see Table 1).

In general, mandibular teeth had more errors as compared to maxillary teeth and this relationship was seen to be statistically significant (*p* = 0.001). The mandibular right quadrant, in particular, showed the most errors (see Table 2). However, there was no significant statistical relationship when right and left teeth were compared (*p* = 0.757).

**Figure 6:**
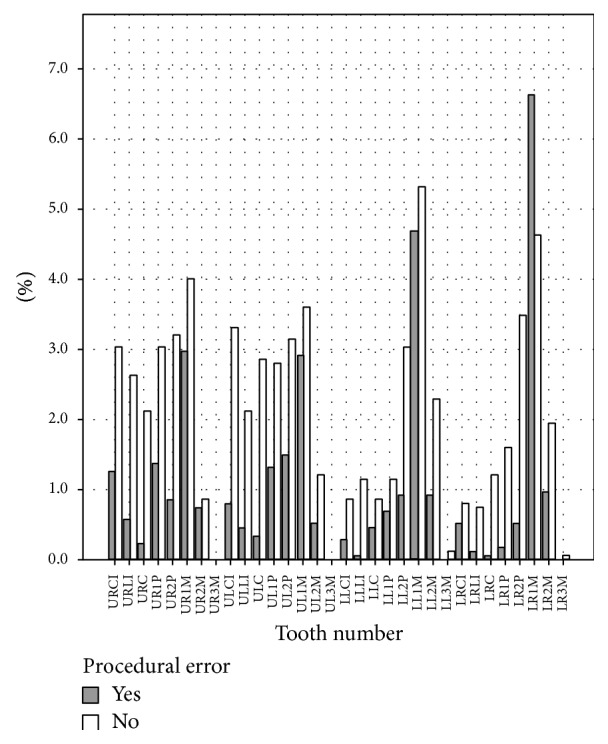
Overall distribution of errors in individual teeth.

The right permanent mandibular first molar was particularly prone to errors, showing a greater overall percentage of errors than any other tooth, and was the only tooth in which errors superseded the acceptable cases (see Figure 6).

**Figure 7:**
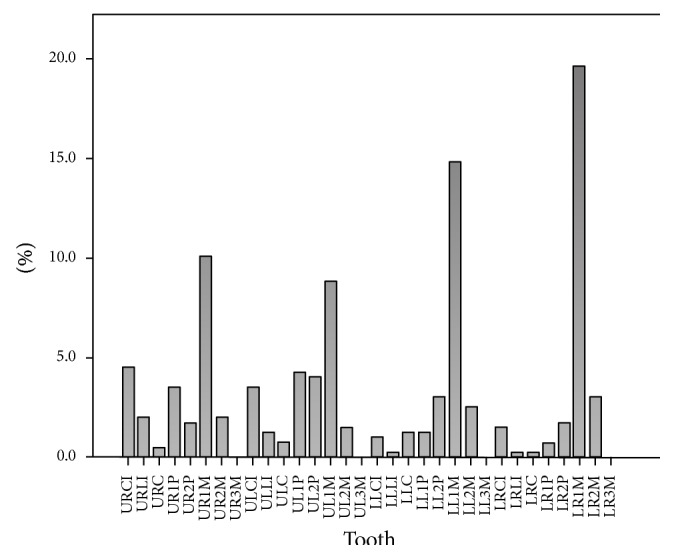
Percentage of overfill in individual teeth.

**Figure 8:**
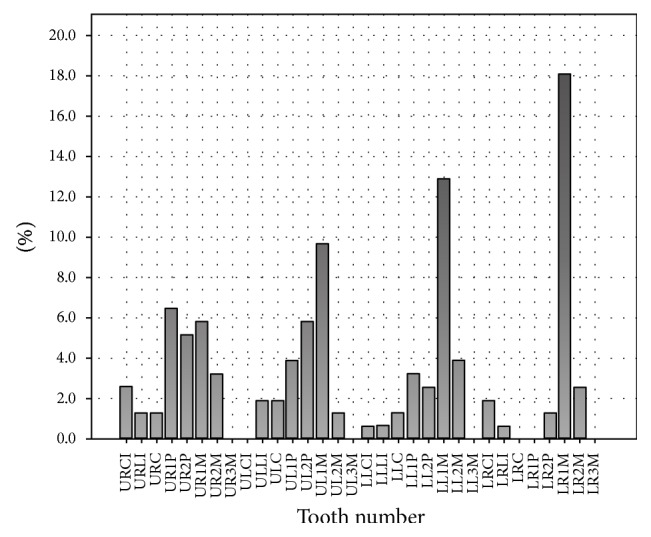
Percentage of underfill in individual teeth.

Percentage of overfill and underfill in individual teeth has been elaborated in Figures 7 and 8.

**Figure 9:**
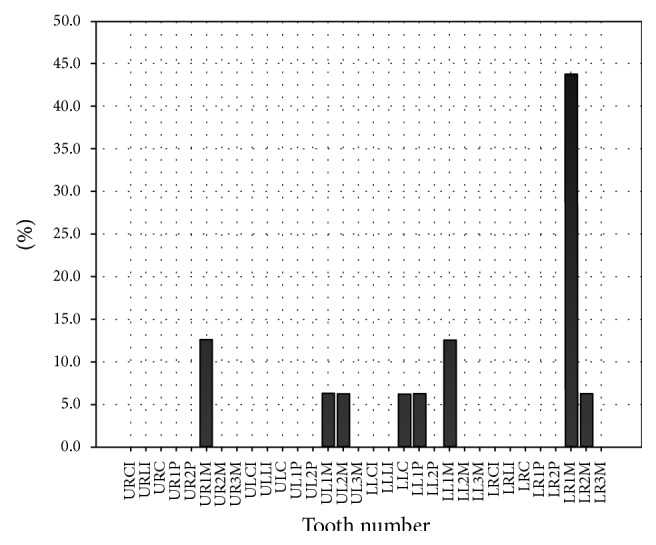
Percentage of instrument separation in individual teeth.

**Figure 10:**
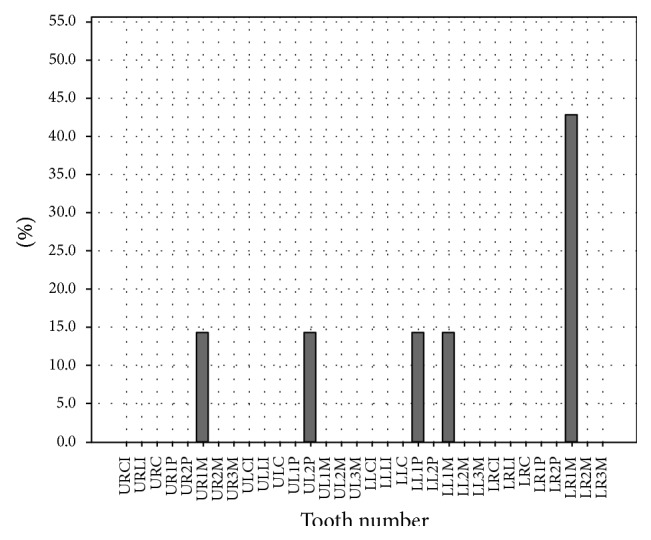
Percentage of apical transportation in individual teeth.

Instrument separation and apical transportation showed the greatest predisposition to the right permanent mandibular first molar (see Figures 9 and 10).

## 4. Discussion

An alarmingly large minority (32.8%) of cases possessed a procedural error. This indicates a need for practitioners to be more meticulous with their technique. Lamentably, at present not enough effort is being made at critical steps during treatment to avoid errors.

**Figure 11:**
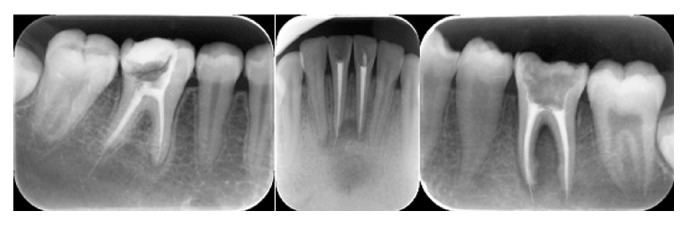
Extrusion of gutta-percha beyond the radiographic apex.

The most common error by far was overfill (22.7%) (see Figure 11). Molars were the largest contributors to this statistic (see Table 1). In particular, mandibular molars had a larger incidence of overfill when compared to their maxillary counterparts. Specifically, the right permanent mandibular first molar was the most susceptible to this error (see Figure 7). The general trend showed that incidence of overfill remains relatively constant in all age groups (after excluding low frequency outlying groups <10 and >60) but was noted to be somewhat higher in the younger age group (10–20 years) (see Table 1). This may be due to inadequate length determination or overinstrumentation [11].

On average molars have the shortest roots [12] as compared to other tooth groups, making them more susceptible to this type of error. Therefore, it is perhaps unsurprising that canines proved to be least affected by this type of error. Incisors and premolars had a similar incidence of overfill (see Table 1). These findings may be attributed to the variations in root morphology present between these different tooth groups, canines having the longest roots [12], making them less susceptible to overfill. Various studies have demonstrated that this procedural accident has a negative effect on the prognosis of overall treatment outcome [7–9]. Although not acceptable, gutta-percha is relatively inert [13] and if extruded beyond the apex has a minimal effect on the healing of the periapical tissues. Conflicting results in numerous studies have made this a controversial topic; therefore, to be on a safe side one should show due diligence and avoid this error altogether.

**Figure 12:**
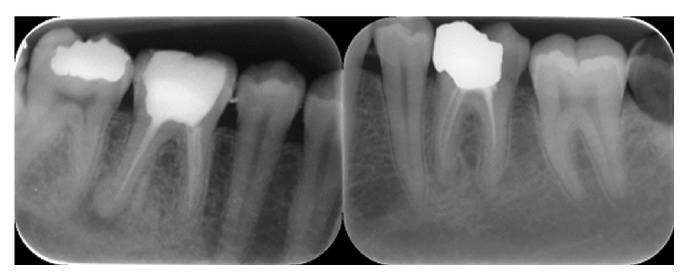
Teeth with gutta-percha more than 2 mm short of the radiographic apex (underfill).

The next most common error was underfill (see Figure 12) which accounted for 8.9% of the total cases. There was little difference in this error when mandibular and maxillary teeth were compared (see Table 2). However, molars were the primary contributors to the rate of error in this category, with the right permanent mandibular first molar being the most affected (see Figure 8). The general trend showed that incidence of underfill was observed to increase with age (after excluding low frequency outlying groups <10 and >60) and was noted to be lower in the younger age group (10–20 years) when compared with the oldest age group (50–60) (see Table 1).

Literature regarding underfill is far clearer in its condemnation and shows the highest failure rates in teeth filled more than 2 mm short of the radiographic apex [14, 15]. This error may be produced by inadequate length determination, inadequate filling technique, use of inflexible files, variations in canal morphology such as excessive curvature and narrow canals (particularly in molars), inadequate irrigation between each filing, and so forth. Furthermore, sclerotic canals and pulp stones may play a role in increased incidence of underfill in the older age group. Unquestionably, all efforts should be made to avoid this type of procedural error.

Instrument separation and apical transportation did not contribute much to the overall percentage of errors observed in our sample (0.9% and 0.4%, resp.). The few cases where instrument separation occurred were more prevalent in the mandible, in particular the right permanent mandibular first molar (see Table 2 and Figure 9). The insignificance of these errors shows that the practitioners are taking adequate steps to avoid such errors.

Analysis of procedural errors when related to individual teeth revealed some intriguing results. Anterior teeth were shown to be significantly less prone to errors than their posterior counterparts. In particular, canines were found to have the least error rate. Interestingly, amongst incisors, central incisors were much more likely to possess an error than lateral incisors. In posterior teeth premolars were found to have less errors compared to molars. Compared to left molars, right molars were more prone to have errors (see Table 2). Most remarkably, the right permanent mandibular first molar was seen to have the highest number of errors in each category (see Figures 7, 8, 9, and 10).

Predictably, permanent mandibular first molars are the most common teeth to undergo endodontic treatment followed by permanent maxillary first molars (see Figure 4). This may be related to their early eruption and favorable morphology (pits and fissures) for plaque retention. This finding is of significant importance as it may show an inability for early detection of a lesion or inadequate prophylaxis on part of the practitioner.

Poor community awareness may also play a role which results in patients reporting to the dentist only when they experience severe pain, leading to progression of the disease process to the extent that endodontic treatment is required. In developing countries like Pakistan patient's low income and lack of education (particularly awareness as regards oral health) act as a barrier to receiving even routine dental checkups. Thus, early detection of any disease process is often not possible and delays preventive treatment, leading to more cases of endodontic treatment. Furthermore, less expertise, lack of specialist practice, and an abundance of roadside quacks also contributed to patient's poor previous dental experience, making them reluctant to seek early dental treatment.

Least common teeth to undergo root canal treatment were third molars (see Figure 4). This is perhaps due to the fact that third molars show the highest degree of morphological variation. This increases the complexity and expertise required for successful treatment. In addition, these teeth often have limited value in mastication/occlusion. Therefore, these teeth are preferentially extracted rather than undergoing endodontic treatment.

Unsurprisingly, mandibular incisors were also amongst the least common endodontically treated teeth. This may be due to smooth labial and lingual surfaces of these teeth which are less susceptible to caries.

## 5. Conclusion

Practitioners should show greater care to maintain accuracy of the working length throughout the procedure, as by far errors in length accounted for the vast majority of errors. Special care should be taken when working on molars, which had a significantly higher error rate when compared to anterior teeth or premolars. Emphasis must be placed on community awareness programs to reduce the incidence of caries progressing to the point of requiring endodontic treatment. High risk patients should be provided with prophylactic treatment (such as fissure sealants and fluoride therapy) and regular routine checkups.

## Figures and Tables

**Table 1:** Distribution of age, gender, and tooth group with endodontic treatment and type of procedural error.

			Type of error					*p* value (*χ* ^2^)
			Normal	Overfill	Underfill	Instrument separation	Apical transportation	
Gender	Male	Count (%)	434 (64.4%)	165 (24.5%)	69 (10.2%)	4 (0.6%)	2 (0.3%)	0.19 (6.12)^*α*^
	Female	Count	726 (68.6%)	230 (21.7%)	86 (8.1%)	12 (1.1%)	5 (0.5%)	

Age group	<10	Count	19 (67.9%)	3 (10.7%)	5 (17.9%)	0 (0%)	1 (3.6%)	0.000139 (57.5)
	10–20	Count	126 (58.1%)	79 (36.4%)	10 (4.6%)	1 (0.5%)	1 (0.5%)	
	20–30	Count	353 (69.9%)	108 (21.4%)	33 (6.5%)	8 (1.6%)	3 (0.6%)	
	30–40	Count	318 (68.2%)	94 (20.2%)	50 (10.7%)	3 (0.6%)	1 (0.2%)	
	40–50	Count	204 (67.5%)	64 (21.2%)	32 (10.6%)	2 (0.7%)	0 (0.0%)	
	50–60	Count	89 (62.7%)	33 (23.2%)	19 (13.4%)	1 (0.7%)	0 (0.0%)	
	>60	Count	54 (71.1%)	14 (18.4%)	6 (7.9%)	1 (1.3%)	1 (1.3%)	

Tooth group	Incisors	Count	256 (78.0%)	57 (17.4%)	15 (4.6%)	0 (0.0%)	0 (0.0%)	0.000 (125.88)
	Canines	Count	123 (86.6%)	11 (7.7%)	7 (4.9%)	1 (0.7%)	0 (0.0%)	
	Premolars	Count	375 (74.6%)	81 (16.1%)	44 (8.7%)	1 (0.2%)	2 (0.4%)	
	Molars	Count	419 (54.1%)	248 (32.0%)	89 (11.5%)	14 (1.8%)	5 (0.6%)	

^*α*^Significance calculated at 95% Confidence Interval (CI).

**Table 2:** Distribution of errors in right and left maxilla and mandible.

	Normal	Overfill	Underfill	Instrument separation	Apical transportation	Total errors
Maxillary right	70.2%	20.6%	8.5%	0.4%	0.2%	29.8%
Maxillary left	70.9%	20.4%	8.1%	0.4%	0.2%	29.1%
Mandibular right	61.7%	26.3%	9.3%	2.0%	0.7%	38.3%
Mandibular left	64.8%	24.1%	9.8%	1.0%	0.5%	35.2%
